# LHCGR phamacodynamic effects during follicular maturation

**DOI:** 10.3389/fendo.2026.1917693

**Published:** 2026-08-12

**Authors:** Carmela Perri, Livio Casarini, Clara Lazzaretti

**Affiliations:** 1 Unit of Endocrinology, Department of Biomedical, Metabolic and Neural Sciences, University of Modena and Reggio Emilia, Modena, Italy; 2 International Ph.D. School in Clinical and Experimental Medicine (CEM), University of Modena and Reggio Emilia, Modena, Italy; 3 Center for Genomic Research, University of Modena and Reggio Emilia, Modena, Italy

**Keywords:** gametogenesis, gonadotropins, hCG, LH, LHCGR

## Abstract

Luteinizing hormone (LH) and human chorionic gonadotropin (hCG) are glycoprotein hormones that regulate human reproduction. Although they bind the same G protein-coupled membrane receptor (LHCGR) and have high percentage of structural identity, recent advances demonstrate that they mediate different signaling pattern according to their separate physiological roles. This review explores the role of LHCGR in determining the evolutionary, genetic, and physiological differences between LH and hCG. Some of these differences were revealed by mutations of the receptors, allowing the identification of LH- and hCG-specific functions in women and men. This knowledge is useful to optimize the use of molecules in clinical practice, aiming toward personalized therapies.

## 1 Introduction

Luteinizing hormone (LH) and human chorionic gonadotropin (hCG) are essential glycoprotein hormones mediating human reproduction and sexual development (1). LH is secreted in a pulsatile manner by the pituitary gland, has a relatively short half-life of approximately 90 minutes, and regulates gametogenesis and androgen synthesis. HCG is the “pregnancy hormone” produced by throphoblast cells and exists in various isoforms and glycoforms with a significantly longer half-life (hours to days) than LH (2). For decades, these two hormones were considered physiologically equivalent because of high percentage of molecular identity (3). They share a common alpha subunit, have a unique beta subunit with approximately 80-85% amino acid identity (4–6), and bind to the same G protein-coupled receptor (GPCR), namely the LH/hCG receptor (LHCGR) (7). Historically, the perceived equivalence, as well as the commercial availability of hCG, led to the widespread clinical use of this molecule as a surrogate for LH activity, particularly in assisted reproduction and the treatment of hypogonadism (3). However, recent scientific advances have disproved the idea that these hormones are fully interchangeable (1). Different molecular features lead to hormone-specific binding to the LHCGR (7, 8), activation of distinct intracellular signaling networks (9), and physiological roles (10). In ovarian cells, LH preferentially activates proliferative and anti-apoptotic kinase pathways, whereas hCG exhibits a high progestational potential (11–13), reflecting its primary role in supporting massive progesterone production during pregnancy (14). The expression levels of the LHCGR itself vary according to the stage of the menstrual cycle (15). In women, it is expressed in theca cells, where the hormone induces androgen production, and in granulosa cells, where it mediates follicle maturation and luteinization (15). In men, LHCGR is exclusively expressed in Leydig cells, as a requisite for LH-induced stimulation of testosterone synthesis (16).

This review explores the evolutionary, genetic, and physiological importance of LHCGR, highlighting how distinct signaling and trafficking signatures underpin the regulation of human fertility.

## 2 The evolution of LH, hCG, and their receptor

The evolutionary history of gonadotropins and their receptors reveals an increasing complexity that mirrors the requirements for refined reproductive strategies in higher vertebrates. Gonadotropins belong to the cystine knot growth factor superfamily, characterized by a common alpha subunit and a hormone-specific beta subunit (4–6, 17, 18). The ancestral gene coding the thyrostimulin beta subunit is thought to have duplicated approximately 927 million years ago, eventually giving rise to the *LHB*, *TSHB*, and *FSHB* genes (19). In primates and equids, a significant evolutionary step occurred with the appearance of chorionic gonadotropins (CG) (20, 21). In the primate lineage, the *CGB* genes putatively evolved through repeated duplications of an ancestral *LHB* gene (22). This process was accompanied by the loss of the original stop codon, resulting in an additional C-terminal extension (CTP) of approximately 30 amino acids in the CG molecule (23, 24). This CTP domain is highly glycosylated, contributing to increase the higher complexity of hCG than LH glycan pattern, consisting in four O-linked and two N-linked glycosylations for the hCG beta subunit, and one N-linked glycan for the LH beta (25). Glycosylation significantly increases the circulatory half-life and bioactivity of hCG compared to LH (26), providing a strategy for sustained progestational support during pregnancy.

**Figure 1:**
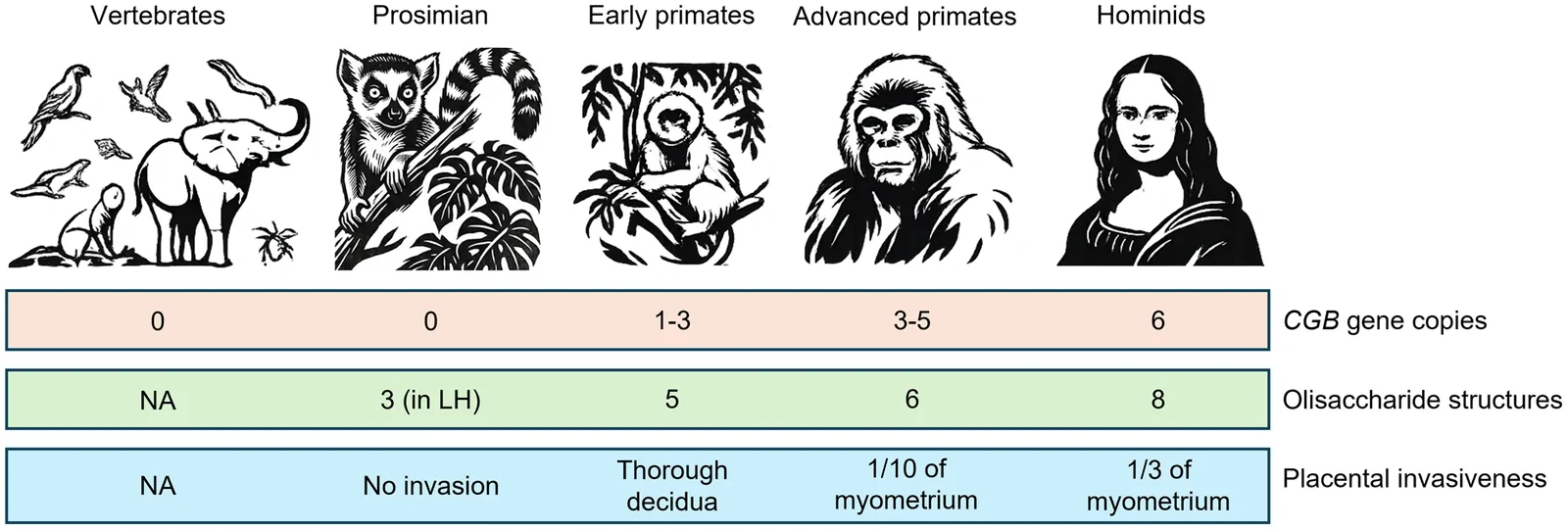
Evolution of the *LHB*/*CGB* system in the vertebrates. Vertebrates have one *LHB* gene, whereas an increasing number of *CGB* genes appears in primates. The complexity of the *LHB*/*CGB locus* increases following the phylogenetic tree from prosimian towards hominids. The number of oligosaccharide structures bound to hormone protein backbones increases as well, along with the placental invasiveness.

In humans, the *LHB*/*CGB* gene cluster on chromosome 19q13.32 has reached its maximum complexity, containing one *LHB* gene and six transcriptionally active *CGB* genes (23). This large number of genes allows for the production of a wide variety of hCG isoforms and glycosylation variants (27). One of the most accredited theories suggests that the rise of *CGB* genes was an evolutionary adaptation to the high energy demands for specialized angiogenic signals required for hemochorial placentation in higher primates (**Figure 1**) (28). Furthermore, it has been hypothesized that these specialized signals evolved to optimize fetal brain development, which should achieve higher complexity in primates than other vertebrates (28). Evolutionary convergence is observed in equids, where pituitary LH and placental CG are products of the same *LHB* gene but differ in their glycosylation patterns (29). These evolutionary diversifications emphasize that the dual ligand system for a single receptor is a refined strategy to manage the conflicting requirements of gametogenesis versus pregnancy.

## 3 The *LHCGR* gene

The human *LHCGR* gene is located on chromosome 2p21, spanning approximately 70–80 kb (30). It encodes a protein of roughly 700 amino acids belonging to the rhodopsin-like GPCR family. The receptor structure is characterized by a large N-terminal extracellular domain containing 11 leucine-rich repeats required for hormone binding, a complex hinge region, and a seven-transmembrane domain (1, 30). A critical feature of the human LHCGR gene is exon 10, which codes a portion of the extracellular hinge region essential for discriminating between the two natural ligands (31, 32). Molecular modeling suggests that while hCG contacts the whole “U-shaped” structure of the hinge region, the smaller LH molecule must specifically interact with a sulfated tyrosine residue (sTyr331) to induce proper receptor activation (8). These concepts could be elucidated using cryo-electron microscopy techniques, which may resolve molecular structures of hormone-receptor complexes. However, only the structure of hCG bound to LHCGR has been reported (33), along with their physical binding features (34), while the tridimensional structure for LH-LHCGR complex is still missing. The fact that all structural data available for hormone binding to LHCGR were obtained using hCG as a ligand suggests that the LH-LHCGR binding structure is hard to be resolved. These difficulties could be likely due to unstable and/or more rapid dissociation (35) from the receptor to LH than hCG, reflected as a faster LH- than hCG-mediated LHCGR internalization-and-recycling in the cell surface (36). In any case, data available converge into the concept that LH and hCG have similar, but not identical interaction sites at the receptor (1, 37). Indeed, naturally occurring deletions of exon 10 result in a receptor that binds both hormones but can only be activated by hCG (38). In men, the exon 10 deletion leads to male hypogonadism because the patient is unresponsive to endogenous pituitary LH, preventing the pubertal development (39). Interestingly, the New World monkey *Callithrix jacchus* lacks a functional LH molecule and has a hCG-like molecule of pituitary origin that supports both gametogenesis and pregnancy, while its receptor naturally lacks the sequence coded by exon 10 (40).

The *LHCGR* gene could also produce several mRNA variants (41). One of them involves a cryptic exon named “6A, “ located between exons 6 and 7, and mutations in this region can lead to Leydig cell hypoplasia type II (42, 43). Another variant lacks exon 9 and results in the receptor being trapped in the endoplasmic reticulum (44). Furthermore, an approximately 50-kDa truncated form of the receptor is supposed to be expressed in the placenta, which might explain the persistent hCG-mediated progestational effect during pregnancy (45). In summary, several *LHCGR* forms were identified at the mRNA level. Except for the “6A” variant, for which consistent data supported its functional impact in human pathophysiology (42, 43), the role of other variants in still unclear and this issue requires further investigation.

Genetic diversity in the *LHCGR* is also represented by single nucleotide polymorphisms (SNPs) and mutations (1, 30). Some of them could be fixed among human populations if they result in mild phenotypes (46). Other SNPs, like the asparagine-to-serine change in the position 312 of the amino acid chain (p.N312S), were associated with spermatogenic damage or polyendocrine metabolic ovarian syndrome risk (47, 48) and may potentially impact the outcomes of ovarian stimulation (49). However, data describing the effects of LHCGR SNPs are still very limited and more studies are needed to know if the p.N312S SNP could be a future target for pharmacogenomic research in assisted reproduction. In contrast, inactivating mutations in the *LHCGR* gene can lead to severe conditions such as Leydig cell hypoplasia type I, characterized by complete lack of masculinization in 46, XY individuals (50, 51). Activating mutations were described as well and can cause male-limited precocious puberty due to sustained tonic production of the second messenger cyclic adenosine monophosphate (cAMP) and androgen synthesis (52, 53).

## 4 LHCGR signaling and trafficking

The binding of LH or hCG to the LHCGR triggers a network of intracellular signaling cascades (9). Both hormones activate the adenylyl cyclase enzyme and subsequent intracellular accumulation of cAMP (13), which is required to induce steroid synthesis (54), although hCG is approximately five times more potent than LH (13, 55, 56). In turn, LH is more potent than hCG in inducing the phosphorylation of extracellularly regulated kinases 1 and 2 (ERK1/2) and protein kinase B (AKT) (13, 36), upregulating directly cell proliferation and survival (11). This phenomenon is an example of biased signaling (57), where different ligands induce different conformational changes in the receptor, favoring specific G protein coupling or recruitment of other intracellular interactors such as beta-arrestins (55). For instance, hCG binding to LHCGR may lead to Gq protein and phospholipase C (PLC) activation, and subsequent intracellular calcium (Ca^2+^) mobilization and inositol-triphosphate (IP3) production, while LH does not (36). In granulosa cells, LHCGR may physically interact with membrane partner molecules, increasing the complexity of the regulation of ligand-induced signals. Some of these partners are the follicle-stimulating hormone (FSH) receptor (FSHR) (58, 59) and the G protein-coupled estrogen receptor (GPER) (60, 61). The latter may form heteromers with LHCGR that selectively bias signaling exerted via allosteric inhibition of the Gq/PLC pathway, but not the canonical Gs/cAMP pathway, preventing intracellular Ca^2+^ increase and reducing cell viability (60). In summary, *in vitro* experiments suggest that LHCGR may form heteromers with other membrane partners, forming functional units linked to unique signaling patterns. Although the impact of heteromers should be further investigated *in vivo*, we may hypothesize that they play a role in fine-tuning gonadotropin signaling during gametogenesis (62).

**Figure 2:**
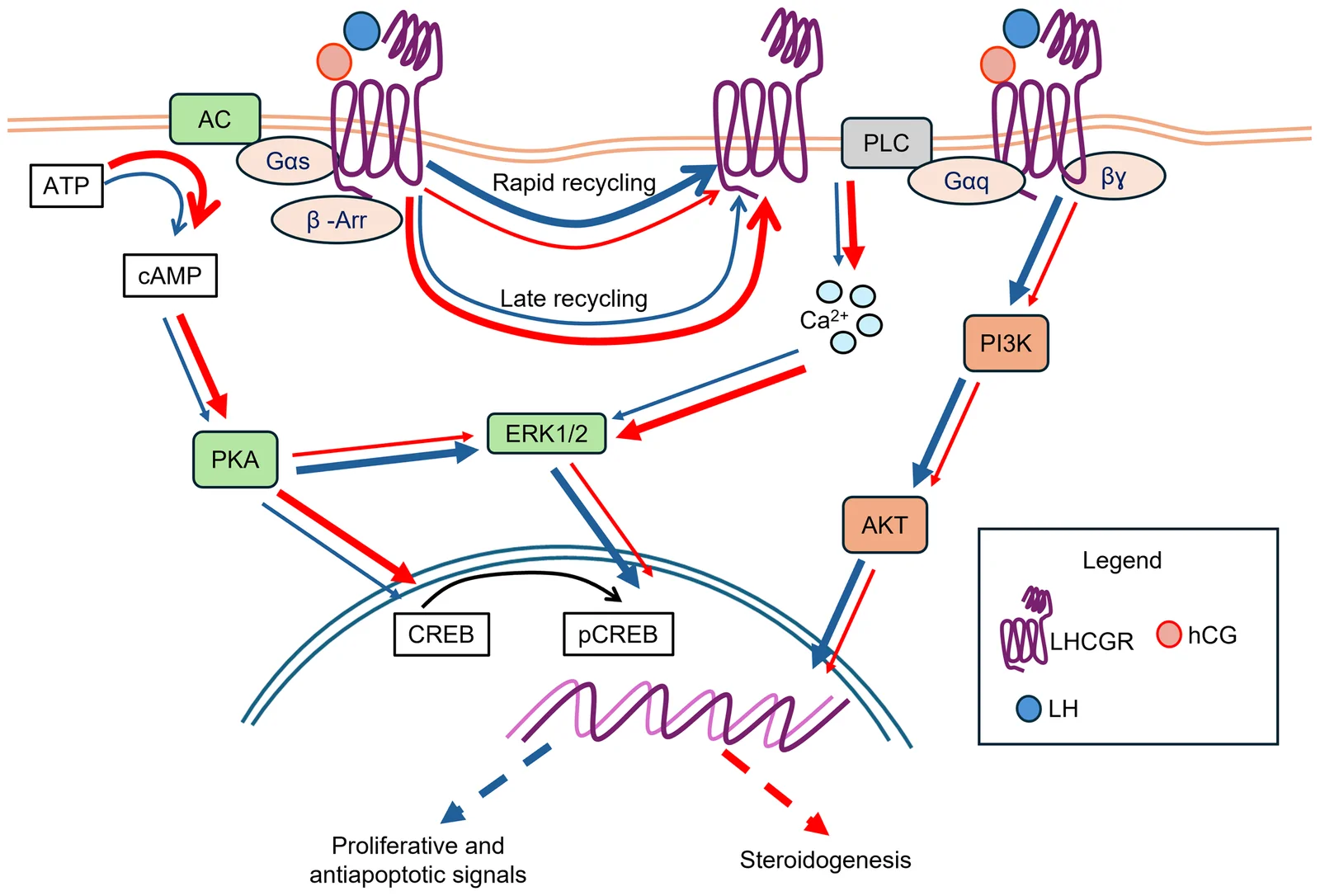
Ligand-dependent LHCGR signaling. Bold lines represent LH- or hCG-preferential activation of the pathway compared to thin lines. LH signaling cascades are showed in blue, whereas hCG signaling in red.

Post-endocytic trafficking of the receptor is another mechanism directing hormone-specific signals (36). Upon ligand binding, the LHCGR is internalized via clathrin-coated pits in a dynamin-dependent manner (63). LH-mediated activation leads to rapid internalization and recycling of the receptor back to the cell membrane through a very early endosomal pathway marked by the adaptor protein, phosphotyrosine interacting with PH domain and leucine zipper 1 (APPL1) (36). This rapid trafficking is essential for maintaining sustained ERK1/2 phosphorylation and LH-induced genomic activity (36), as well as the maintenance of LH-induced cAMP production (64). In contrast, hCG-induced trafficking is slower and involves more robust beta-arrestin 2 recruitment, which sorts the receptor into early and late endosomes positive to the Ras-related protein 5 (Rab5) and 7 (Rab7), respectively (36). This prolonged compartmentalization in endosomes is likely required to support the persistent steroidogenic signals needed during pregnancy, since the blockade of LHCGR internalization impaired cAMP production (36). The different trafficking signatures of LH and hCG provide a location bias that allows the same receptor to regulate different physiological processes, such as mitogenic support of gametogenesis for LH and sustained steroidogenesis for hCG (36). Altogether, these data suggest revisiting the historical concept of LHCGR desensitization (65, 66). This concept was proposed to explain the time-dependent loss of adenylyl cyclase activity detectable in gonadal cells treated by LHCGR agonist (67). Current approaches for signaling studies are more sensitive than old methods based on radioactive compounds and allows detection of previously unknown signaling cascades. Therefore, a more recent view of the kinetics of GPCR functioning depicts temporally, spatially and perhaps functionally distinct waves of ligand-induced signaling (68), which are still active after internalization (**Figure 2**).

## 5 Role of the LHCGR in the antral follicular stage

In women, the ovarian follicle is the functional unit supporting oocyte growth, maturation and ovulation, and these functions are orchestrated by FSH and LH (69). According to the “two cells-two gonadotropins” theory, LH targets theca cells to stimulate androgen production, which then serves as a substrate for FSH-driven estrogen synthesis in granulosa cells (70). However, LHCGR also plays a role in granulosa cells throughout the antral stage, where it mediates fine-tuned mitogenic and anti-apoptotic signals (71).

**Figure 3:**
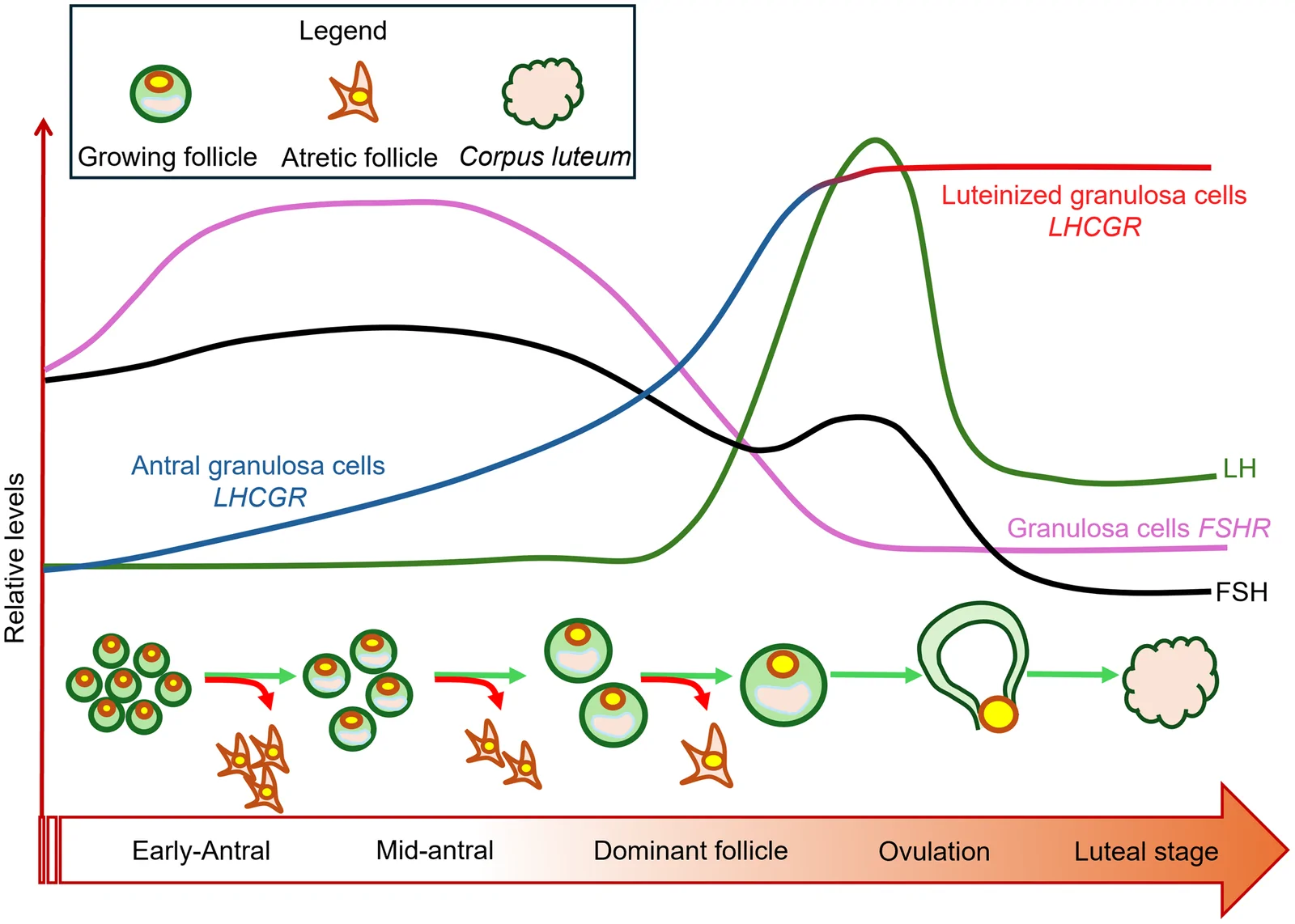
Representative picture for *LHCGR* and *FSHR* expression levels throughout the menstrual cycle.

During the early antral stage, the expression of the *LHCGR* gene is relatively low (**Figure 3**), while *FSHR* expression is at its peak (15). At this stage, it was traditionally believed that a low percentage (<1%) of LHCGR molecules behave as “spare” receptors, allowing for maximal androgen production despite low receptor occupancy (72). Recent evidence suggests an alternative model, based on the concept of FSHR-LHCGR heterodimerization, better depicting the events mediating early antral androgen production (62). In granulosa cells, these receptors form functional complexes where FSH binding to the FSHR can trigger “LH-like” proliferative signals through the LHCGR (9, 62, 71, 73). This cooperation is likely essential for driving follicular progression when LH levels are relatively low, since androgens are the substrate for estrogen production (74), as a requisite for granulosa cell proliferation, and follicle growth and maturation (75). Estrogen synthesis is induced by FSH, which triggers the cAMP-pathway activation and expression of the aromatase enzyme-coding gene (76). Persistently high intracellular cAMP levels may lead to pro-apoptotic events (77–79) possibly linked to granulosa cell collapse and follicular *atresia* (61). The progress of the antral stage is characterized by interactions between the FSHR and GPER, which could be responsible for dominant follicle survival (61). These two receptors form heteromers that reprogram FSH-induced cAMP signals into AKT-dependent survival stimuli (61). This mechanism is thought to be fundamental in rescuing the dominant follicle from *atresia* while others in the cohort, characterized by a higher FSHR: GPER ratio and excessive cAMP activity, undergo follicular collapse (61). As the follicle matures toward the late antral and pre-ovulatory stages, LHCGR expression progressively increases in granulosa cells, likely via FSH-dependent mechanisms (80–82), replacing FSHR (15). This shift is necessary to switch the follicle from a growth-oriented phase to one prepared for ovulation and luteinization (83). High levels of LH activity at mid-cycle induce the production of epiregulin and amphiregulin, which are EGF-like ligands essential for oocyte maturation and follicle rupture (84). Studies in mouse preovulatory follicles suggested that LH exerts these effects via activation of Ca^2+^ signaling (85), although this hypothesis requires further studies in human cells since *in vitro* data revealed the failure of LH-induced Ca^2+^ increase (36). Following ovulation, LHCGR mediates the differentiation of granulosa into luteal cells, initiating the preferential progesterone production required to support a potential pregnancy. This is a function transiently supported by LH action in the *corpus luteum* and it is destined to cease within about two weeks in the event of failure to conceive.

## 6 Role of LHCGR in pregnancy

The progression of human pregnancy is dependent on the sustained activation of the LHCGR in the *corpus luteum* (86). During the first 7 to 10 weeks of gestation, pituitary LH levels decline due to negative feedback from rising steroid levels, and its luteotropic role is replaced by hCG of trophoblastic origin. HCG is secreted in a constantly increasing fashion, peaking in the first trimester, continuously sustaining the stimulation of progesterone production (86).

Beyond its progestational role, hCG is essential for placentation and angiogenesis (87). It was hypothesized that these functions could be mediated by specific hCG variants, such as hyperglycosylated hCG molecules (hCG-H) (88). HCG-H is predominantly produced in early pregnancy by extravillous cytotrophoblasts and likely acts as an autocrine factor to promote trophoblast invasion into the maternal decidua (89). Insufficient levels of hCG-H are predictive of miscarriage (90, 91), highlighting its role as a master regulator of early embryo development. It was supposed that hCG-H binds to transforming growth factor beta receptor II (TGFβRII) (92), instead of LHCGR, although this view has been experimentally challenged (93) and more studies are required to elucidate the mechanism of action of the hyperglycosylated hormone.

**Figure 4:**
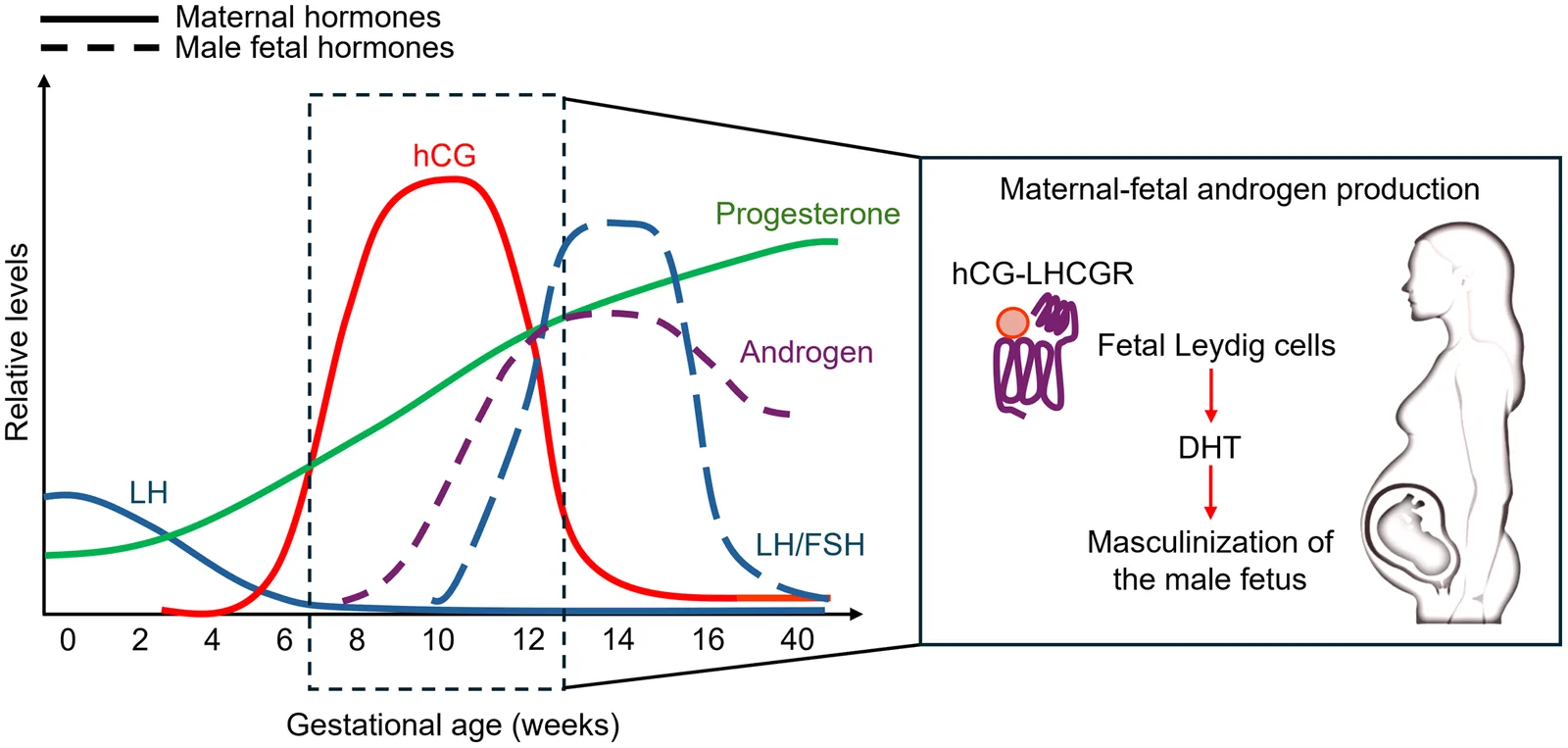
Variations of maternal and fetal hormone levels during pregnancy. HCG peak corresponds to increasing maternal progesterone and fetal androgen levels, while the gonadotropin release by fetal pituitary occurs later. The development of fetal Leydig cells by hCG leads to activation of DHT synthesis via the backdoor steroidogenic pathway.

HCG also stimulates maternal androgen production (74, 94). It is required for fetal hypothalamus-pituitary-gonadal (HPG) axis development and sex steroid production (**Figure 4**), and has long-term effects on the child’s future fertility (95). In male fetuses, hCG triggers the masculinization of external genitalia by stimulating dihydrotestosterone (DHT) synthesis (96). During this stage, DHT is produced via the “backdoor” pathway for androgen synthesis, which is an alternative route for synthesizing the steroid without passing through testosterone (96). Disruption of the enzymes driving this pathway can result in moderate to severe undervirilization in male babies, or hyperandrogenism in female fetuses (96, 97). Moreover, hCG exerts a TSH-like action by cross-activating the TSH receptor (98, 99), which helps sustaining maternal thyroid hormone production during the first trimester (100). In summary, LHCGR and hCG are two of the primary mediators of the complex endocrine dialogue between the mother and the developing fetus.

## 7 Physiology of LH in the male

In the male, LH is the primary driver of testicular function and secondary sexual development (101). LH targets LHCGR expressed exclusively on Leydig cells located in the interstitial compartment of the testis (102). The primary physiological response to LH stimulation is the synthesis of testosterone from cholesterol (74). Unlike in the female ovary, where cells must discriminate between proliferative and steroidogenic signals, Leydig cells appear to respond to LH and hCG with a qualitatively similar balance of cAMP and kinase signaling (56). Testosterone produced by Leydig cells acts in a paracrine manner on Sertoli cells to support spermatogenesis (103), although some of it is converted to estradiol to promote germ cell survival (104). The secretion of LH by the pituitary is controlled by hypothalamic GnRH in a pulsatile fashion, as a requirement for maintaining testosterone production at physiological levels (105). After birth, a transient surge in pituitary LH and FSH occurs (106). This period is known as minipuberty and results in a surge of testosterone similar to pubertal levels, and it is relevant for early testicular development (106).

The importance of functional LH signaling in males is starkly demonstrated by genetic defects. Inactivating mutations in the *LHB* gene result in hypogonadotropic hypogonadism, characterized by delayed puberty, small testes, and low testosterone (107). However, because the LHCGR remains functional, these individuals can achieve virilization, testicular growth, and even fertility through long-term treatment with exogenous hCG. In contrast, inactivating mutations of the LHCGR lead to much more severe phenotypes, such as Leydig cell hypoplasia type I (108). These 46, XY individuals are phenotypically female at birth due to a severe impairment of testosterone-driven masculinization during fetal development (109). This difference demonstrates that maternal hCG may compensate for a lack of fetal LH during gestation if the receptor is intact, whereas proper pituitary LH production is mandatory for masculinization and secondary sex characteristics after birth.

## 8 Clinical use of LH and hCG

In clinical practice, gonadotropins are most frequently used for controlled ovarian stimulation (COS) as part of assisted reproduction technologies (ART) (110). Historically, urinary hCG was the only available source of supposed “LH activity” because it was easily purified from the urine of pregnant women (111, 112). These considerations are at the basis for long lasting use of hCG as it would be interchangeable with LH to support follicular growth (113), even if they are not biologically identical (1). With the advent of recombinant DNA technology, both LH and hCG preparations have become available for clinical use, offering greater purity and batch-to-batch consistency and the possibility to compare effects of the two hormones in a clinical setting (114). Recently, it was characterized that intrafollicular levels of LH rapidly increases and peaks between 12–17 hours, before decreasing, after pituitary stimulation by GnRH agonist, while intrafollicular hCG peaks between 32–36 hours (115). These profiles are suggestive of ligand-specific kinetics of receptor activation inside the follicle and are consistent with the existence of rapid and fine-tuned regulation of intracellular kinases by LH, as well as with sustained support to the steroidogenic pathway exerted by hCG (36). In fact, hCG addition to controlled ovarian stimulation leads to slightly higher androgen levels than FSH alone (115), Instead, a meta-analysis suggested that the addition of LH may improve oocyte quality and pregnancy rates per oocyte number (116). This is consistent with *in vitro* data showing that LH induces direct proliferative and anti-apoptotic signals in granulosa cells (11, 13), and supports the concept that LH supplementation could be recommended for poor responders and women of advanced maternal age (117–119). However, comparisons between the two hormones need relatively large sample size to appreciate differences in the clinical outcomes, and it is hardly achievable by single case-control studies. After the follicle growth, controlled ovarian stimulation needs the trigger of final oocyte maturation and ovulation (120). While LH is the physiological trigger, hCG is traditionally used because of its longer half-life (121) and historical availability (122), although other strategies, such as GnRH agonist administration (123), may be used decreasing risk of ovarian hyperstimulation syndrome (OHSS). In the case where GnRH agonist trigger is used, appropriate luteal support with progesterone must be applied (124), as the GnRH agonist-induced LH surge is weaker than the physiological one (125). Whereas GnRH agonists retain the potential to trigger both LH and FSH surge as in the natural cycle, the hCG-induced trigger does not (115), although the role of FSH surge at the mid-cycle is unknown. Also hCG may be used for luteal phase support, following trigger by agonist, to improve luteal function and progesterone output (126). In summary, LH and hCG in ART may be used according to the specific needs of the patient, and after an evaluation aiming to optimizing oocyte yield, quality, and minimizing the risk of adverse events.

In men, the primary clinical application of hCG is the treatment of hypogonadotropic hypogonadism (HH) (127, 128). Because HH is characterized by the lack of endogenous pituitary gonadotropins, the administration of hCG effectively restores eugonadism by stimulating Leydig cells to produce testosterone. Convincing evidence exists for its efficacy in inducing virilization, increasing testicular volume, and initiating spermatogenesis to restore fertility in combination with FSH (129, 130). Interestingly, an ongoing clinical trial is proposing that recombinant LH may also be effective in treating male HH (131), and it has been suggested that LH, in combination with FSH, may be sufficient to restore testosterone levels, possibly replacing hCG administration (132). Taken together, these data suggest a possible clinical employment of LH that warrants further investigation, as the use of this molecule in men is currently not permitted by most regulatory agencies.

HCG has been used for a long time for pharmacological treatment of cryptorchidism, where the hormone may induce testicular descent (133). However, this treatment has been associated with germ cell apoptosis and DNA fragmentation, potentially impacting reproductive function in adulthood (134). On one hand, this effect could be consistent with the pro-apoptotic potential observed when cells are exposed to high levels of cAMP signaling induced by hCG (11). Therefore, it could be hypothesized that using LH instead of hCG might dampen these effects. However, clinical guidelines recommend shifting from hormonal therapy in favor of surgical orchidopexy as the standard of care to preserve future fertility (135). On the other hand, the impact of hCG on sperm DNA fragmentation should be further tested in a clinical setting. The only major adverse events to consider during hCG therapy in men are related to excessive serum testosterone levels, which can lead to negative effects on the liver, red blood cells, and the prostate gland (136). In summary, hCG is a standard of care for restoring male reproductive function, but future clinical trials with LH could offer greater insight for new therapeutic alternatives.

## 9 Conclusions

LH and hCG are distinct ligands for the LHCGR, exhibiting different signaling and trafficking patterns, leading to hormone-specific intracellular signatures likely evolved to optimize human gametogenesis in both the sexes *versus* pregnancy in women. Despite these differences, only hCG has been used for several decades in the treatment of male and female infertility, while knowledge on the mode of action of LH has increased in recent times. These studies elucidated molecular mechanisms distinguishing the action of both molecules, establishing the basis for optimizing their clinical use.
